# Definition of the Mediterranean Diet: A Literature Review

**DOI:** 10.3390/nu7115459

**Published:** 2015-11-05

**Authors:** Courtney Davis, Janet Bryan, Jonathan Hodgson, Karen Murphy

**Affiliations:** 1 Alliance for Research in Exercise, Nutrition and Activity, University of South Australia, Adelaide 5001, Australia; courtney.davis@mymail.unisa.edu.au; 2 School of Psychology, Social Work and Social Policy, University of South Australia, Adelaide 5001, Australia; janet.bryan@unisa.edu.au; 3 School of Medicine and Pharmacology, University of Western Australia, Crawley 6009, Australia; jonathan.hodgson@uwa.edu.au

**Keywords:** Mediterranean diet, definition, quantity, foods and nutrients

## Abstract

Numerous studies over several decades suggest that following the Mediterranean diet (MedDiet) can reduce the risk of cardiovascular disease and cancer, and improve cognitive health. However, there are inconsistencies among methods used for evaluating and defining the MedDiet. Through a review of the literature, we aimed to quantitatively define the MedDiet by food groups and nutrients. Databases PubMed, MEDLINE, Science Direct, Academic Search Premier and the University of South Australia Library Catalogue were searched. Articles were included if they defined the MedDiet in at least two of the following ways: (1) general descriptive definitions; (2) diet pyramids/numbers of servings of key foods; (3) grams of key foods/food groups; and (4) nutrient and flavonoid content. Quantity of key foods and nutrient content was recorded and the mean was calculated. The MedDiet contained three to nine serves of vegetables, half to two serves of fruit, one to 13 serves of cereals and up to eight serves of olive oil daily. It contained approximately 9300 kJ, 37% as total fat, 18% as monounsaturated and 9% as saturated, and 33 g of fibre per day. Our results provide a defined nutrient content and range of servings for the MedDiet based on past and current literature. More detailed reporting amongst studies could refine the definition further.

## 1. Introduction

The Mediterranean diet (MedDiet) was first defined by Ancel Keys as being low in saturated fat and high in vegetable oils, observed in Greece and Southern Italy during the 1960s [1]. In the Seven Countries Study this dietary pattern was associated with reduced risk of coronary heart disease (CHD) compared to northern European countries and the United States after 25 years follow-up [2,3]. Over the past several decades the study of the MedDiet has advanced, and the definition originally introduced by Keys has evolved and varied. There are a number of ways to define a dietary pattern, including general descriptions, dietary pyramids, *a priori* scoring systems, *a posteriori* dietary pattern formation, or by food and nutrient content [4,5,6,7,8,9].

Of these, *a priori* scoring systems have gained most popularity in the past two decades as they simplify analysis of adherence to the diet in relation to primary outcomes [10]. Dietary intake is separated into selected food groups related to health outcomes and points are awarded for higher intakes of health-promoting foods and lower intakes of health-harming foods, to calculate a single adherence score. However there are several *a priori* Mediterranean diet scores (MDS) with different scoring criteria [11,12,13,14]. Sofi *et al.* [10] recently compared data from 26 cohort studies utilising some form of MDS, and noted the large range of cut-offs for major food groups such as cereals, even amongst similar populations. When compared on the same nutritional data, 10 different *a priori* MDS resulted in a mean adherence ranging from 22.7% to 87.7%, with poor correlation between most indices [14]. This implies the defining aspects of the MedDiet used to calculate these scores are widely different. Similarly, there are large differences between studies using gram intakes of foods and nutrient content as descriptions/adherence scores. For example, the Greeks in the Seven Countries study consumed an average of 191 g/day of vegetables, in the Prevención con Dieta Mediterránea (PREDIMED) study participants in the intervention group consumed approximately 350 g/day, and the Greeks enrolled in EPIC consumed over 500 g/day [11,15,16].

Marinez-Gonzalez *et al.* [1] suggest that “the very definition of the MedDiet is not a minor issue” (p 10), and point out that two prominent randomised trials investigating health effects of the MedDiet used interventions not fully in line with traditional ideas of the diet, such as the high oil content. A systematic review of intervention trials investigated the relationship between the MedDiet and health outcomes; the authors concluded that there is good evidence the diet improves the lipid profile, endothelial function and blood pressure, but that one of the most limiting factors to drawing conclusions was discrepancies in how the MedDiet had been defined and formulated [17].

Differences in definitions could be limiting our understanding of the mechanisms by which the MedDiet confers its health benefits. The biological actions of key nutritional components of the MedDiet, such as specific fatty acids, have been studied with promising, although somewhat inconsistent results [18]. One reason for this may be differences in dose of foods and nutrients between studies. Additionally it is difficult to formulate new MedDiets for intervention studies which are consistent with previous studies, as there is little consistency on which to base these new diets. One potential approach to address such problems is to form a more universal definition by calculating an average quantity of foods and nutrients from previous MedDiets, which would combine traditional and modern examples from relevant studies and provide a benchmark profile of the MedDiet. This definition could be used in future to design intervention MedDiets or MDS which are comparable to other studies. Our objective was to collate information from a range of studies to form a more comprehensive and quantitative definition of the MedDiet than presently exists, by summarising existing definitions and calculating the mean amounts of foods and nutrients.

## 2. Results

### 2.1. Mediterranean Diet: General Descriptions and Pyramids

General descriptions of the MedDiet are similar amongst publications, emphasising the same key components. The definitions include guidelines for high intake of extra virgin (cold pressed) olive oil, vegetables including leafy green vegetables, fruits, cereals, nuts and pulses/legumes, moderate intakes of fish and other meat, dairy products and red wine, and low intakes of eggs and sweets. Each description provides an indication of the frequency these foods should be consumed, for example often, daily, biweekly and the amounts in the diet, described using subjective terms such as abundance, high, moderate, low, some, and vast. Most lack specific suggestions for numbers of servings or serving size, and do not specify amounts of additives to the diet, such as sauces, condiments, tea, coffee, salt, sugar, or honey. Some definitions specify that cereals should be mostly wholegrain. The definitions from Willett *et al.* [6] Panagiotakos *et al.* [19] and Dilis *et al.* [20] add some description of traditional practices; olive oil was added to vegetables and legumes to make them palatable, fruits were eaten as desserts or snacks, cheeses accompanied salads and stews, and red meat was eaten only on special occasions.

Most commonly, recommended numbers of servings for these food groups are represented as a diet pyramid. Diet pyramids are considered a useful way to display the general principles of a diet including approximate recommendations for quantities of food groups (*i.e.*, those consumed in greatest quantities appear in the largest section of the pyramid). Three MedDiet pyramids were chosen as a representative sample for this review, although several others exist. In 1993, the first MedDiet pyramid was produced by Oldway’s Preservation and Exchange Trust [6]. This was updated in 2009 [21]. The 1999 Greek Dietary guidelines are based on a traditional MedDiet and are also expressed in pyramid form [22]. A third pyramid model of the diet was released in 2010 by the Mediterranean Diet Foundation (MDF), intended as a flexible, general representation of the MedDiet [5]. The pyramid of the Greek dietary guidelines is semi-quantitative, providing serving number and size. The recommendations of these three pyramids are compared in Table 1.

**Table 1:** Comparison of dietary recommendations for three Mediterranean diet pyramids.

Foods	Oldway’s Preservation and Trust (2009) [21]	Mediterranean Diet Foundation (2011) [5]	1999 Greek Dietary Guidelines (1999) [22] ^1^
Olive oil	Every meal	Every meal	Main added lipid
Vegetables	Every meal	≥2 serves every meal	6 serves daily
Fruits	Every meal	1–2 serves every meal	3 serves daily
Breads and cereals	Every meal	1–2 serves every meal	8 serves daily
Legumes	Every meal	≥2 serves weekly	3–4 serves weekly
Nuts	Every meal	1–2 serves daily	3–4 serves weekly
Fish/Seafood	Often, at least two times per week	≥2 serves weekly	5–6 servings weekly
Eggs	Moderate portions, daily to weekly	2–4 serves weekly	3 servings weekly
Poultry	Moderate portions, daily to weekly	2 serves weekly	4 servings weekly
Dairy foods	Moderate portions, daily to weekly	2 serves daily	2 serves daily
Red meat	Less often	<2 serves/week	4 servings monthly
Sweets	Less often	<2 serves/week	3 servings weekly
Red wine	In moderation	In moderation and respecting social beliefs	Daily in moderation

^1^ Serving sizes specified as: 25 g bread, 100 g potato, 50–60 g cooked pasta, 100 g vegetables, 80 g apple, 60 g banana, 100 g orange, 200 g melon, 30 g grapes, 1 cup milk or yoghurt, 1 egg, 60 g meat, 100 g cooked dry beans.

The general structure and placement of key food groups in the pyramids is similar however the pyramids differ in their recommendations for vegetables and fruits, nuts and legumes, fish/seafood and poultry. Recommendations for legume intake range from every meal to at least twice a week. The MDF suggests daily nuts, while the Greek guidelines are less specific and recommend fewer servings.

### 2.2. Mediterranean Diet: Quantity of Food in Grams

The gram quantity for foods or food groups was reported for 15 separate populations in 11 papers, spanning a timeline of 46 years of data collection (1960–2006) [8,11,16,23,24,25,26,27,28,29,30]. Twelve of the 15 reports were based on observations of dietary intake, most commonly collected by food frequency questionnaire or food recalls [8,11,16,23,24,26,27,28]. All of these observations were of local Mediterranean populations excepting one, which observed Greek-Australian migrants living in Melbourne, Australia [26]. Of the three interventions, two were in a local French Mediterranean population, the third in an Australian population [25,29,30]. All reports included gram values for the groups all cereals, all vegetables, fruits/nuts and meat/meat products. All dairy and legumes were reported for all but one population and fish in all but two. Reporting of bread, potato, cheese, eggs and oil was less consistent; olive oil was reported in 11 data sets, eggs in nine, cheese and potato in seven and bread intake recorded in five. Table 2 shows the amount of foods in grams in the MedDiet by study, and the average and standard deviation for each food group. Table 3 shows the conversion of grams of foods to standard Australian serving sizes, compared to recommendations according to the Australian Dietary Guidelines [19]. According to the lowest and highest intakes reported, the MedDiet contained between three and nine serves of vegetables, half to two serves of fruits, one to 13 serves of cereals and 1.5 to eight serves of olive oil daily. The recommended number of servings according to the Greek Dietary Guidelines and the MDF pyramid differ considerably from the average numbers of servings derived here, based on Australian serving sizes. Fruits, nuts and fish servings are considerably less than recommended.

### 2.3. Mediterranean Diet: Nutrient Content

Eight papers reported the nutrient content of the MedDiet in sufficient detail to be included in this review [4,15,27,28,29,31,32,33]. Table 4 shows the mean nutrient content of the MedDiet. Four of the eight included papers were intervention studies, two were descriptive papers and two were observational studies. The interventions took place in France, Australia, Spain and Sweden [15,25,29,33]. The observations were of Spanish adults recruited for the European Prospective Investigation into Cancer and Nutrition (EPIC) study [27,28]. The two descriptive papers analysed the nutrient content of Mediterranean menus designed specifically for the study, both based on the traditional Cretan diet [4,32]. Two others reported only on specific fatty acid content of the diet (Table 5) [3,11].

All papers reported the energy and fibre content, and all but one reported the per cent energy contribution from saturated fat (SFA). It was possible to derive the monounsaturated fat (MUFA) to SFA ratio for all papers. According to these eight papers, the MedDiet contains approximately 9.3 MJ/day, and provides close to 37% energy from total fat, 19% from MUFA, 5% from PUFA, 9% from SFA, 15% from protein and 43% energy from carbohydrate. The specific fatty acids intakes observed from the Seven Countries Study and amongst the Greek cohort of the EPIC study are shown in Table 5 [3]. The addition of this data did not greatly alter the average total fat and energy contents, although they did result in slightly higher intakes. Two papers included long chain omega-3 PUFA content expressed as % of total energy, with an average of 1.4%, and three papers as grams/day, with an average of 1.1 [4,15,25,27,33].

### 2.4. Mediterranean Diet: Flavonoid Content

Bioactive compounds include a range of non-nutritive substances thought to confer health benefits, including polyphenolic compounds and phytosterols [34]. The most notable of the polyphenols are flavonoids, water soluble plant components which are known to have antioxidant properties *in vitro* [34]. There are six classes of flavonoids including flavones, flavonols, flavanols (flavan-3ols), flavanones, anthocyanidins and isoflavones [35]. These are sourced primarily from red wine, olive oil, coffee, tea, nuts, fruits, vegetables, herbs and spices. Four papers reported specifically on the flavonoid content of the diet [21,36,37,38]. The mean daily flavonoid content presented in the four papers is compared in Table 6. Vasilopoulou *et al.* [38] developed a theoretical seven-day traditional Mediterranean menu based on the 1999 Greek dietary guidelines [39]. The 2003 United States Department of Agriculture (USDA) flavonoid tables were used to estimate flavone, flavonol, flavanol, flavanone and anthocyanidin content, and isoflavone content was estimated from the VENUS phytoestrogen database [38]. Dilis *et al.* [21] analysed this same menu chemically for the luteolin, apigenin (flavones), quercetin, kaempferol, myricetin, isorhamnetin (flavonols) and catechin, epicatechin, epigallocatechin, epicatechin gallate and epigallocatechin (flavanols) content and determined the total daily content as 79.01 mg. Vasilopoulou *et al.* [38] found the combined total of flavones, flavonols and flavanols was 67.8 mg/day, as calculated from the 2003 USDA tables (Table 6). Zamora-Ros *et al.* [36] using an updated USDA flavonoid database (2007) estimated the total daily flavonoid intake of the Spanish cohort recruited for the EPIC study. Dietary intake was assessed by a computerised diet history questionnaire administered by a dietitian in a personal interview. Tresserra-Rimbau *et al.* [37] conducted the only analysis of polyphenol intake based partially on an intervention MedDiet. Over 7000 high-risk Spanish adult participants with complete dietary data were included from the PREDIMED study. Polyphenol intake, including flavonoids, was calculated using the Phenol-Explorer database which provides information on polyphenol content for 456 foods. Dietary data was collected from a 137-item FFQ, administered in person by a dietitian.

**Table 2:** Grams per day of key foods and food groups in the Mediterranean diet, in order of date data was collected, including mean ± SD.

			Food Groups											
Reference	Study Type/Notes	Years of Data Collection	Bread	All Cereals ^1^	Legumes	Potato	All Vegetables ^2^	Fruits/Nuts	Meat/Meat Products ^3^	Cheese	All Dairy ^4^	Eggs	Olive Oil	Fish ^5^
Alberti-Fidanza and Fidanza (2004) [39] ^6^	Observational study, Italian cohort, 1960s. Based on WFRs. Male adults only	1960	NR	488	49	NR	344	101	53	15	48	20	NR	42
Alberti-Fidanza and Fidanza (2004) [39]	Observational study, Italian cohort, 1960s. Based on WFRs. Female adults only	1960	NR	348	36	NR	274	76	29	12	49	11	NR	30
Kromhout *et al.*, (1989) [16]	Observational study, Greek cohort 1960s. Based on 7-d WFRs	1960–1965	415	452.5	30	170	361	463	35	13.5	165.5	15	80	39
Kromhout *et al.*, (1989) [16]	Observational study, Italian cohort, 1960s. Based on 7-d WFRs	1960–1969	353	513	5.5	43	210	109.5	119.5	28.5	223.5	46.5	60.5	28.5
Varela-Moreiras *et al.*, (2010) [23] ^6,7^	Observational study, Spanish cohort, based on FFQs	1964	368	434	20.2	300	451	155	77	NR	∼229	NR	∼68	63
Trichopoulou *et al.*, (1995) [8] ^8^	Observational study, Greek cohort. Based on FFQs	1988–1990	NR	288	60.5	NR	286	264	112.5	NR	246	NR	NR	NR
de Logeril *et al.*, (1994) [24]	Intervention study. MedDiet *vs.* advice from hospital dietitians. Based on 24-h recalls	1988–1992	167	261	19.9	NR	316	251	105.0	32.2	NR	NR	15.7	46.5
Kouris-Blazos *et al.*, (1999) [25]	Observational study, Greek-Australians. Based on FFQs	1990–1992	NR	353	86	NR	252	246	190	NR	246	NR	NR	NR
Guallar-Castillon *et al.*, (2012) [26]	Observational study, Spanish middle-age adults. Based on FFQs	1992–1996	NR	230	52	79	334	325	126	27	297	26	20	63
Buckland *et al.*, (2009) [27] ^9^	Observational study, Spanish adults. Based on FFQs	1992–1996	NR	198.2	52.2	NR	269.3	353.3	128.0	NR	323.8	NR	20.7	61.1
Trichopoulou *et al.*, (2003) [11]	Observational study, Greek cohort. Based on FFQs. Males only.	1994–1999	NR	191.0	10.4	98.9	682.5	393.0	129.3	NR	222.6	19.0	46.2	26.4
Trichopoulou *et al.*, (2003) [11]	Observational study, Greek cohort. Based on FFQs. Females only.	1994–1999	NR	145.7	7.9	73.5	609.6	385.7	94.9	NR	216.2	15.7	38.9	21.7
Itsiopoulos *et al.*, (2011) [28]	Intervention study MedDiet *vs.* HabDiet. Based on 7-day diet records	1998–2001	190	263	56	116	582	307	45	25	126	19	65	37
Vincent-Baudry *et al.*, (2005) [29]	Intervention study. MedDiet *vs.* low fat diet. Based on 3-d WFR	1998–2002	NR	200	NR	NR	350	303	150	NR	200	NR	20	100
Varela-Moreiras *et al.*, (2010) [23] ^6^	Observational study, Spanish cohort. Based on FFQs	2000–2006	NR	221.1	12.6	NR	302.1	298.6	182.0	NR	398.1	34.7	49.5	98.4
		**Mean**	298.6	305.8	35.6	125.8	374.9	268.7	105.1	21.9	213.6	23.0	44.0	50.5
		**SD**	112.3	118.5	24.3	86.5	142.0	115.0	49.9	8.2	96.2	11.2	22.7	25.6

^1^ All cereals group includes all refined and whole grain products reported, including bread; ^2^ All vegetables including potatoes; ^3^ Meat/Meat products group includes all unprocessed and processed red meat, white meat and delicatessen meats unless otherwise specified; ^4^ All dairy includes cheese, milk and milk products, and yoghurt as reported; ^5^ Fish includes oily fish, non-oily fish and shellfish; ^6^ Fruit and nuts group includes fruit only; ^7^ Meat/meat products includes chicken only; ^8^ Original data presented as mean daily consumption in grams adjusted for energy, separated into survivors and dead. Average presented; ^9^ Intakes divided into tertiles of MedDiet score, average of three tertiles used. Mean of both genders reported. Originally reported as g/1000 Calories, converted to total by (grams*(total Calories/1000)). WFR, weighed food record; NR, not reported; FFQ, food frequency questionnaire.

**Table 3:** Servings of food groups in the Mediterranean diet, calculated as standard Australian serving sizes, compared to Australian Dietary Guidelines [30], Greek Dietary Guidelines [22] and Mediterranean Diet Foundation [5] recommendations.

	Food Groups												
	Bread	All Cereals	Legumes ^1^	Potato	All Vegetables	Fruits ^2^	Nuts ^1,2^	Meat/Meat Products ^1^	Cheese	Other Dairy	Eggs ^1^	Olive Oil	Fish ^1^
Average content of the MedDiet, g/day ^3^	300	305	35	125	375	225	4	105	21	215	23	45	50
Australian standard serving size, g or mL [30]	40	40–120	75–150	75	75	150	30	65–80	40	200–250 g	120	10 mL	100
Number of standard serves	7.5/day	2.5–7.5/day (average 4)	0.25–0.5/day, 1.6–3/week	1.5/day, 11.5/week	5/day	1.5/day	1/week	0.5–0.75/day	0.5/day, 3.5/week	1/day	0.25/day, 1.75/week	4.5/day, 31.5/week	0.5/day, 3.5/week
ADG recommended serves/day, adult men	NS	6	3	NS	6	2	3	3	NS	2.5	3	NS	3
ADG recommended serves/day, adult women	NS	6	2.5	NS	5	2	2.5	2.5	NS	2.5	2.5	NS	2.5
GDG recommended serves	NS	8/day	3–4/week	NS	6/day	3/day	3–4/week	Red meat 4/month, Poultry 4/week	NS	2/day	3/week	Daily, NFS	5–6/week
MDF recommended serves	NS	1–2/meal	>2/week	<3/week	>2/meal	1–2/meal	1–2/day	Red meat <2/week, White meat 2/week	NS	2/day	2–4/week	Every meal, NFS	>2/week

^1^ Lean meat/poultry/fish/eggs/nuts and seeds/legumes grouped together in dietary guidelines, no recommendation for number of serves for individual foods such as nuts, legumes, fish or seeds; ^2^ Fruits and nut intakes based on papers provided this information (8 papers reported fruit, 2 papers reported nuts). Mean combined fruits/nuts was 270 g calculated in Table 2; ^3^ rounded to nearest 5 g. ADG, Australian Dietary Guidelines; GDG, Greek Dietary Guidelines; NS, not specified; NFS, not further specified; MDF, Mediterranean diet foundation.

**Table 4:** Daily energy and nutrient content of the Mediterranean diet, in order of date of data collection, including mean ± SD.

			Nutrients and Energy																
Reference	Study Type/Notes	Years of Data Collection	Energy ^1^ (kJ/kCal)	Total Fat (g)	PRO (g)	MUFA (g)	PUFA (g)	SFA (g)	% E from Total Fat	% E from MUFA	% E from PUFA	% E from SFA	MUFA: SFA Ratio ^2^	% E from CHO	% E from Protein	Fibre (g)	Vit C (mg)	Folate (μg)	Pot (mg)
Kafatos *et al.*, (2000) [4].	Descriptive study. Based on WFR	1960–1965	11016/2633	77	NR	67	18	25	41.3	23	6.0	9	2.68	45	12	47	258	559	4504
de Lorgeril *et al.*, (1994) [31].^3^	Intervention study. Based on 24-h food recall and FFQ	1988–1992	8146/1947	NR	NR	NR	NR	NR	30.4	12.9	4.6	8	1.6	NR	16.2	18.6	115.8	NR	NR
Buckland *et al.*, (2009) [27].^4^	Observational cohort study. Based on FFQs	1992–1996	8669/2072	82.5	90.75	37.5	11.8	22.6	35.2	16	5.1	9.6	1.66	40.8	17.8	27.4	172.7	NR	NR
Gualllar-Castillon *et al.*, (2012) [26].^5^	Observational cohort study. Based on FFQs	1992–1996	10021/2395	NR	110.2	NR	NR	NR	NR	NR	NR	NR	2.00	NR	NR	25.3	279.9	512.5	NR
Trichopoulou *et al.*, (2006) [32].	Descriptive study. Based on menu designed from GDG	1999 ^6^	2473/10347	110.7	74.5	63.8	9.9	29.8	39.6	22.8	3.5	10.7	2.14	39.6	12.2	29.8	NR	NR	1774
Itsiopoulos *et al.*, (2011) [28].	Intervention study. Based on 7-day diet records	1998–2001	9300/2223	NR	NR	NR	NR	NR	NR	21.3	NR	8.2	2.60	43.5	13.5	36.2	191.1	453	4565
Ambring *et al.*, (2004) [33].	Cross over intervention. Based on 24-h recalls.	NR	7820/1869	NR	NR	NR	NR	NR	NR	14	NR	8	1.75	48	16	40	NR	NR	NR
Estruch *et al.*, (2013) [15] ^7^	Intervention study. Based on FFQs	2003–2010	9205/2200	NR	NR	NR	NR	NR	NR	21.5	NR	9.4	2.04	40	16.3	26.2	NR	NR	NR
		**Mean**	9316/2226	89.9	91.8	56.3	13.3	25.9	36.6	18.8	4.8	9.0	2.1	42.8	14.9	31.3	203.5	508.2	3614.3
		**SD**	1101/236	18.2	17.9	15.9	4.2	3.5	4.9	4.3	1.0	1.0	0.4	3.3	2.3	9.2	66.3	53.1	1594.1

^1^ Where energy reported in kilojoules, converted to Calories (kilojoules/4.184). To convert from Calories to kilojoules, multiply by 4.184; ^2^ If not provided, calculated from grams fat. If grams not provided, calculated from percentage energy from MUFA and SFA; ^3^ Dietary intake from experimental group presented, at an average of 4 years follow-up. Vitamin C presented as ascorbic acid from original publication (June 1994), de Lorgeril *et al.* [24]. Percent energy from MUFA presented as oleic acid only (18:1 omega-9); ^4^ Reported mean intakes of highest tertile for MedDiet adherence. Reported as g/1000 Calories. Converted to total grams by formula (grams × (Calories/1000)). Calculated energy contributions (%) from grams of macronutrients; ^5^ Highest quintile reported for MedDiet adherence score. PRO and total fat reported as g/1000 Calories. Converted to total grams by formula (grams × (Calories/1000)); ^6^ Based on 1999 Greek dietary guidelines; ^7^ Average of walnut and olive oil intervention groups presented. PRO, protein; MUFA, monounsaturated fat; PUFA, polyunsaturated fat; SFA, saturated fat; CHO, carbohydrate; Vit, vitamin; mg, milligrams; μg, micrograms; POT, potassium; WFR, weighed food record; NR, Not reported; FFQ, food frequency questionnaire; GDG, Greek dietary guidelines; SD, standard deviation.

**Table 5:** Mean nutrient intake (grams per day) reported in the Seven Countries Study (Greek and Italian cohorts) and EPIC study (Greek cohort).

	Nutrients and Energy, Mean ± SD				
	Energy (kJ/kCal)	SFA (g)	MUFA (g)	PUFA (g)	MUFA:SFA
Kromhout *et al.*, (1995) [3]. (Greeks)	2749 ± 100	24.9 ± 4.4	73.7 ± 14.7	14.4 ± 1.3	3.0 ± 0.1
Kromhout *et al.*, (1995) [3]. (Italians)	3043 ± 523	38.0 ± 14.1	58.9 ± 12.3	15.3 ± 5.5	1.6 ± 0.3
Trichopoulou *et al.*, (2003) [11]. (Greek Males)	2438 ± 705	34.6 ± 13.2	58.4 ± 20.0	17.5 ± 9.2	1.8 ± 0.5
Trichopoulou *et al.*, (2003) [11]. (Greek Females)	1931 ± 572	28.6 ± 11.6	48.7 ± 17.8	15 ± 8.2	1.7 ± 0.5
Mean ± SD (Table 5 only)	10628.5 ± 1989.2/2540 ± 475	31.5 ± 5.9	59.9 ± 10.3	15.6 ± 1.4	2.0 ± 0.7
Mean ± SD (Table 4 and Table 5)	9753.2 ± 1506.2/2331 ± 360	29.1 ± 5.5	58.4 ± 11.9	14.6 ± 2.9	2.0 ± 0.5

EPIC, European Prospective Investigation into Cancer and Nutrition; SFA, saturated fat; MUFA, monounsaturated fat; PUFA, polyunsaturated fat; MUFA:SFA, monounsaturated to saturated fat ratio.

**Table 6:** Comparison of mean daily flavonoid intakes of the Mediterranean diet.

	Study Type	Years Data Collected	Participant Characteristics	Total Daily Flavonoid Intake (Mean) mg/Day
Zamora-Ros *et al.*, (2010) [34]	Observational study	1992–1996	Spanish adults aged 35–64	313.3
Tresserra-Rimbau *et al.*, (2014) [35]	Intervention study. MedDiet administered to half study sample	2003–2010	Spanish adults aged 55–80	Quintiles from lowest to highest: 273 362 431 512 670
Vasilopoulou *et al.*, (2005) [36]	Descriptive	NA	NA	118.6
Dilis *et al.*, (2007) [20] ^1^	Descriptive	NA	NA	79.1
Average flavonoid content				344.9

^1^ Dilis *et al.* [20] included only flavones, flavonols, and flavan-3ols (flavanols) in their analysis; NA = not applicable.

## 3. Discussion

The MedDiet has been described similarly for the past five decades, and several pyramids represent the general principles. However, this review found that studies vary considerably when defining the amounts of foods in grams and/or nutrients constituting the MedDiet, although less so when the nutrient profiles are compared.

Dietary constituents of the MedDiet may reduce the risk of CVD and cancer in a dose dependent manner, highlighting the need for greater consistency between studies in the amount of foods and nutrients administered as part of a MedDiet. Sofi *et al.* [37] reviewed the dietary data of the Greek component of the EPIC study, and using segmented logistic regression models evaluated the dose-response relationship between intakes of the nine components of the MDS and overall mortality. There appeared to be an increased risk reduction at two threshold levels for intakes of fruits and nuts, meat and meat products, ethanol, vegetables, cereals and dairy [37].

Evidence from the present study shows considerable variation in quantity of MedDiet components. The intake of olive oil ranged from 15.7 to 80 mL/day, legumes from 5.5 to 60.5 g/day, vegetables from 210 to 682 g/day and fruits and nuts from 109 to 463 g/day amongst studies. A 5-fold difference in olive oil intake and 10-fold difference in legume intake could have significant implications for specific and all-cause mortality risk. Menotti *et al.* [2] used Seven Countries Study data to examine whether modest variations in food intake predicted changes in CHD death rate. The daily increase for oils (30 g), legumes (30 g), all vegetables (+ 20%, 189 g) and all vegetable food (+ 25%, 237 g) all predicted decreased death from CHD (by 18%, 28%, 28%, and 32%, respectively). Amongst the Spanish population of the EPIC study, for each 10 g increase in olive oil, the hazard ratio was 0.93 for risk of all-cause mortality (95% CI 0.90–0.97) [38]. Furthermore, sub-analyses from the PREDIMED study showed after 3 months on the intervention, C-reactive protein was significantly decreased in the extra virgin olive oil-enriched arm, but not the nut-enriched arm [40]. After 12 months, a 24 g/day increase in extra virgin olive oil resulted in a 0.3 μg/L decrease in TNF-α receptor 60 concentration, and a 62.7 g/day increase in vegetable intake resulted in a 0.2 μg/L decrease (*p* < 0.05) [41]. The variety of olive oil intake alone seen across different studies could affect whether the study finds significant effects of the MedDiet.

Quantity of foods appears to impact health outcomes, and forms the basis for most *a priori* MDS scoring criteria [11]. Meta-analytic evidence has shown those consuming more vegetables, fruits/nuts, legumes, cereals and fish, less dairy and meat/poultry and who have a higher MUFA:SFA and consume moderate amounts of ethanol have better cardiovascular and cognitive health than those consuming less [42,43]. However, the quantity used to define cut-offs varies between studies; when the 9-point MDS score was first used in 1995 the cut-off for vegetable intake was 303 g/day for men, and when used again in 2003 this increased to 550 g/day [8,11]. Differences of such magnitude are likely to substantially alter intakes of bioactive nutrients. Furthermore any subtle improvements in health with increasing intakes may be lost when only one cut-off point is used. There have been recent attempts to improve these scores—Sofi *et al.* [10,37] in their work have proposed scores with multiple cut-offs and using weighted mean cut-offs from a number of studies. While these newer scores are probably improvements on existing MDS’s, they are still limited by a number of factors, such as failing to recognise major foods like nuts, and differences between studies as to foods are included into each food group. An average nutrient content may be more useful as a basis for forming *a priori* scoring systems.

In this review, nutrient content was found to be more consistent across different studies than food quantity. Different foods can provide similar nutrients which allows for preservation of unique foods and dishes observed amongst the different Mediterranean countries while retaining the mechanistic effects of the nutrients and bioactive compounds. Thus there is a distinct advantage to defining the diet by nutrients rather than foods. There are currently no *a priori* based scores which use nutrient content exclusively [11,19]. Consumption of fatty acids as a percentage of total energy intake, protein, the MUFA to SFA ratio and fibre, vitamins C and E, minerals including selenium and potassium, folate, β-carotene, antioxidant or phytosterol content may be useful nutrients to consider in defining the diet, as these nutrients are consistently implicated as combining for anti-CVD, anti-cancer, anti-aging effects and preventing cognitive decline [36,42,44,45,46]. According to this review, on average PUFA intake contributed 4.9% total energy, MUFA 18.4%, SFA 9.0%, the MUFA:SFA was 2.0, fibre intake was 33 g/day, vitamin C 225 mg/day and folate 508.2 μg/day. Notably, it was not possible to derive detailed information on the content of nutrients such as selenium, vitamin E, beta-carotene, long chain omega-3 PUFA or other bioactives such as plant sterols. Expressing nutrient intake as a percentage of total energy is recommended, as those consuming more energy will usually consume more nutrients [47].

Previously, Sauro-Calixto *et al.*, formed a definition of the MedDiet based on nutrient intakes of the Spanish population in 1964 [9]. This definition focused only on four biologically active components of the diet; fibre, total daily antioxidant capacity, MUFA:SFA and phytosterol content. The MUFA:SFA suggested to define the diet was 1.6–2.0, compared to 2.0 in the present study. This appears to be a consistent element of the MedDiet. A defining dietary fibre intake of 41–62 g/day compares to an average intake of 33 g/day found in the present study, with a large variation amongst both interventions and observations [15,16]. It is possible that fibre intake has been too low in recent interventions. The other two components of this definition are rarely considered in studies, total daily antioxidant capacity and phytosterol intake. From the four studies included in the review investigating the total flavonoid content, intake is likely to be at least 79 mg/day with an average of approximately 350 mg. Estimates for flavonoid intakes ranged from 79 to 670 mg/day, depending on population studied and whether chemical analysis or databases were used. Indeed there are so many methods for determination of flavonoids that it is not possible to compare studies. Standardization of practices for determination of flavonoids is necessary before we can accurately compare different MedDiets and calculate an approximate range or average [15,24].

Because servings of foods tend to be better received than nutrients or grams in public health, we calculated the number of standard Australian serves provided on average by the MedDiet [30]. Based on the average gram content, the MedDiet provides approximately seven serves of bread, four serves of cereals, five serves of vegetables, 1.5 serves of potato, 1.5 serves of fruit, 0.5–0.75 serves of meat, 0.5 a serve of cheese, and one serve of dairy per day, as well as one serve nuts and three serves of legumes and fish per week. Popular Mediterranean pyramids recommend at least 3–4 weekly serves of nuts, and at least three daily serves of fruit, and usually fewer serves of potato. Considering the averages were based primarily on observation studies these difference are understandable—there appears to be a mismatch between the reality of what Mediterranean populations are eating and pyramid definitions of the MedDiet. This is one limitation of this review, which did not attempt to distinguish between definitions of the MedDiet based on whether they came from observations of diet, or intervention diets.

This review was limited by several other factors. Limited reporting of key nutrient or bioactive molecules has already been mentioned. Only four of 12 food groups had gram values from all 15 data sets. Only energy and fibre was provided by all eight studies, and seven provided the per cent contribution to total energy intake from the macronutrients. Three reported amounts of fats, protein and carbohydrates in grams, and only four reported on other key nutrients including calcium, potassium, phosphate, magnesium, sodium, folate and vitamins A, E and C. There was rarely information provided on sugar, sources of sugar (e.g., desserts or sweets) or wild greens and other herbs, known sources of antioxidants. The average values must be interpreted with some caution.

There were inconsistencies in classification of food groups. For example, the fruit and nuts group consisted only of fruits for intakes reported by Varela-Moreiras *et al.* [23] and Alberti-Fidanza *et al.* [39]. Potentially, separation of fruits and nuts would be worthwhile, as nuts appear to have an independent role in health [48]. Little information was given on the diet formation when administered as an intervention. It was often unclear whether there was consideration for origin of the diet, which (if any) previous research it had been modelled on, and where and how foods were sourced.

It may be becoming increasingly important to distinguish between observed modern MedDiets and the traditional model based primarily on the Cretan, Greek and Southern Italian diets of 1960 and prior, as countries move towards a more Westernised eating pattern. Most pyramids and general descriptions are still based on traditional practices. However the intervention diets included in the review were in some cases “inspired” by the MedDiet but had distinct differences to typical traditional diets. Ambring *et al.* [33] formulated an intervention diet with less calories and total fat than the control diet, despite the traditional MedDiet typically being high in energy and moderate to high in total fat. Several of the observations were of modern MedDiets, for example Spain in the early 2000s [23]. If the uniqueness of the traditional diet is lost, the longevity and protection against CHD observed in the Seven Countries Study may also be lost. However, arguably it is prudent to include modernised MedDiets in the definition, which incorporate new health research and allow for changes in food supply and habits, such as was done in the formation of the MDF pyramid [5]. It may no longer be possible to follow a traditional diet for most populations, especially outside of Mediterranean countries.

## 4. Materials and Methods

To define the MedDiet a literature review was performed. Databases PubMed [49], MEDLINE [50], Science Direct [51], Academic Search Premier [52] and the University of South Australia Library Catalogue [53] were searched using the following search terms; “Mediterranean diet”, “Mediterranean dietary pattern”, Mediterranean, and content, nutrient *, “nutrient content”, definition, define *, pyramid, and “number of serve *”, flavonoid *. Definitions were classified into one of four categories for the purpose of this review: (1) general descriptive definitions; (2) diet pyramids/numbers of servings of key foods and serving size; (3) grams of key foods/food groups; and (4) nutrient and flavonoid content. Total intakes of other phytochemicals, including total polyphenols, phytosterols and carotenoids, were reported by *n* < 2 papers which was deemed insufficient information to draw conclusions from. Papers were included in the review if they defined the MedDiet in at least two of the above four ways. This included studies reporting the dietary intake of a Mediterranean population in an observational capacity, presenting a Mediterranean menu designed based on evidence, or studies using a Mediterranean diet as an intervention where the grams of foods and/or nutrient content was reported. All studies were published in English. There was no restriction on study design, date of publication or sample characteristics. MedDiets used for weight loss purpose were excluded due to caloric and food restrictions. Studies reporting less than five nutrients or food group quantities were excluded. Where the same MDS had been applied using identical cut-off values in separate articles, only the original paper was included.

To define the diet by quantity of foods in grams or milllitres, the mean intake was recorded for major foods or food groups. Twelve groups were included (bread, all cereals, legumes, potatoes, all vegetables, fruits/nuts, meat/meat products, cheese, all dairy, eggs, olive oil and fish), based on available data. For the all cereals, all vegetables and all dairy groups, if not originally reported the sum of individual components was used; for all cereals, bread and cereals were combined, for all vegetables, potatoes and other vegetables were combined and for all dairy, cheese, yoghurt and milk were combined. Using standard Australian serving sizes [30] the gram value was converted to numbers of serves to provide a defining range or number of servings for key food groups. To define the diet by nutrient intake, the mean of all studies reporting at least five of the following was calculated; energy, total fat, SFA, MUFA, protein, percentage energy contributions from total fat, SFA, MUFA, PUFA, protein, carbohydrate, MUFA:SFA, fibre, vitamin C, folate and potassium. The percentage energy contributions of macronutrients and the MUFA to SFA ratio were calculated based on total gram value where not originally reported.

## 5. Conclusions

High level evidence from the PREDIMED [15] study shows the MedDiet can improve cardiovascular and cognitive health, which will help guide dietary guideline development for prevention of chronic disease. To help understand the mechanisms of benefit of the MedDiet, we require a definition of the MedDiet which is consistent and describes not only the principles generally, but the nutrient and bioactive content. This is the first review to collate information from across a wide range of different studies, to summarise the general descriptions, servings of foods, grams of key food groups and nutrient and flavonoid content of the MedDiet. The average nutrient content of the diet resulting from this review was relatively consistent amongst different studies. This may be a useful working definition, and could improve *a priori* scoring systems which currently rely on grams of foods rather than nutrient content. Consideration for the geographical location of data collection, sample size, and methodological aspects was beyond the scope of this review, but may be necessary to improve the definition. Examining additional foods, nutrients and bioactive compounds and including an assessment of methodological quality of studies could improve this definition further.

## Supplementary Materials

Supplementary materials can be accessed at: http://www.mdpi.com/2072-6643/7/11/5459/s1, Table S1: Study design, objectives, methods, outcomes and details of the MedDiet for each paper included in the review, Table S2: Full list of nutrient content presented by studies defining the MedDiet, intakes presented as total per day.
