# Supplementary material for: Definition of the Mediterranean Diet: A Literature Review
Source: Nutrients. 2015 Nov 5;7(11):9139–53. doi: 10.3390/nu7115459 (PMC4663587; doi:10.3390/nu7115459)
Supplement: Supplementary file 1 [file nutrients-07-05459-s001.docx]

**Supplementary Materials**

**Table S1**. Study design, objectives, methods, outcomes and details of the MedDiet for each paper included in the review.

| **Reference** | **Design** | **Aim/Primary Objective** | **Population/Years Follow-up** | **Methodology** | **Details of the MedDiet** | **Outcomes** |
| --- | --- | --- | --- | --- | --- | --- |
| Studies Reporting Both Nutrient Content And Grams of Foods | | | | | | |
| de Lorgeril *et al.*, (1994) [1] | Randomised, controlled,  single-blinded secondary prevention trial | To compare the effect of a Mediterranean alpha-linolenic acid-rich diet to the usual post-infarct prudent diet on secondary cardiac mortality and morbidity. | French adult CVD patients (mean age 53.5 years, only  those aged <70 were eligible, mean BMI 25.8 kg/m^2^)/1988–1992 | Myocardial infarction survivors were randomised to either a Mediterranean style diet or hospital dietitian advice only (control group). An intermediate follow-up of at least 1 year was preformed after which point the trial was stopped due to clinically significant differences performed at baseline and at 8 weeks follow-up, then annually through a 24 h recall and FFQ. | More bread, root and green vegetables, fish, less meat (especially red meat), fruit daily and use of olive oil and a canola-oil based margarine with a similar composition to olive oil as the main added fats. Moderate consumption of red wine with meals allowed. Nutrient intake and grams of foods recorded at baseline, 8 weeks, 52 week sand 104 weeks for the experimental group. | Cholesterol, triglycerides, apoproteins and lipoprotein, weight and blood pressure were similar between groups at 104 weeks follow up. Those in the MedDiet group had a 70% ↓ risk of mortality (risk ratio 0.30%, 95% CI 0.11–0.83, *p* = 0.02), and a 73% ↓ risk of fatal and non-fatal myocardial infarction and death from other cardiovascular causes (risk ratio 0.27%, 95% CI 0.12–0.59, *p* = 0.001) after 27 months follow-up. |
| Buckland *et al.*, (2009) [2] | Prospective  cohort study | To prospectively investigate the relation between adherence to a MedDiet and incident CHD events within the EPIC-Spain cohort. | Healthy Spanish adults (M = 15,632,  F = 25,806, age  29–69 years,  mean BMI 28.3 kg/m^2^)/1992–2004 | Sociodemographic and dietary data collected at baseline and 3 year follow-up. Dietary data collected through a computerised dietary history questionnaire. End points non-fatal and fatal coronary events determined at 3-year follow-up and by hospital discharge records, mortality registries and myocardial infarction registries over a mean follow-up of 10 years. | A relative MedDiet score used to assess adherence. The intakes of 9 key components were divided into tertiles (per 1000 kcal/day). Values 0, 1 and 2 were assigned to the tertiles, positively scoring for fruit, vegetables, legumes, cereals, fish and olive oil, negatively scoring for meat and dairy. For ethanol, 2 points were awarded for intakes between 5–25 g/day for F and 10–50 g/day for M, and 0 points for intakes outside these ranges. | Adjusted analysis; risk of CHD ↓ by 40% (95% CI 0.47–0.77,  *p* < 0.05) between highest and lowest MedDiet adherence groups. 1-unit increased in score associated with a 6% (95% CI 0.91–0.97, *p* < 0.05)  ↓ risk. |
| Varela-Moreiras *et al.*, (2010) [3] | Cross-sectional study | To assess Spanish population food availability per capita per day,  to then | Randomly selected adults from Spanish population/ | The sample was selected in two stages—first, local entities or towns were selected, and in the second stage, households were | The MedDiet of the modern Spanish population is described. The diet of the Spanish population in  1964 is also | The modern Spanish diet is high in vegetables, fruit, fish and seafood, alcohol and oils and fats and is moderate in |
|  |  | calculate energy and nutrient  intake enabling  a comparison with the recommended nutrient intakes. | 2000–2006 | randomly selected from within the entities. 6000 homes were selected for the years 2000–2005, and 8200 for the year 2006. Households completed the FCS, detailing the shopping and product entry to the home. From this quantity of foods (g/day/person) and nutrients  were calculated using food composition tables. | described and compared  with modern intakes to show changes over the past  40 years. | cereals, dairy, pulses and legumes, meat and meat products. It remains low in precooked foods and sugar and sweets. Macronutrient contributions to energy have changed since 1964- fat contributed 32%, proteins 12%, carbohydrates 53% and alcohol 4% to total energy in 1964, while in 2006 41% of energy came from fat, 14% from protein, 41% from carbohydrates and 5% from alcohol. Fruit, dairy and meat consumption has increased since 1964 and vegetable and greens and cereal intake has decreased markedly. |
| Itsiopoulos *et al.*, (2011) [4] | Randomised  cross over controlled trial. | To investigate the impact of a diet modelled on the traditional Cretan MedDiet on metabolic control and vascular risk in type 2 diabetes. | Australian Type 2 Diabetic patients (M = 16, F = 11, age range 47–77 years, mean BMI 30.7 kg/m^2^)/ 1998–2001 | Participants randomised to consume a MedDiet for 12 weeks and their habitual diet for 12 weeks, in a cross-over design (no wash-out). Participants completed 7-day WFRs at baseline and at the end of each dietary period. Fasting blood samples collected at baseline and end of each period (week 12 and 24). | Intervention diet was based on the traditional Cretan diet, reconstructed from the SCS WFRs and traditional recipe books from the 1960’s. The majority (70%) of the diet was provided to participants as pre-prepared foods. | HbA1c significantly reduced after following the intervention diet, compared to control (↓ 0.3%, *p* = 0.012). There were no significant changes in BMI, fasting glucose, fasting insulin, HOMA score, total, LDL and HDL cholesterol, triglycerides, blood pressure, CRP, homocysteine or the albumin to creatinine ratio. |
| Guallar-Castillon *et al*., (2012) [5] | Prospective cohort study. | To analyse data from the EPIC study to examine the association between *a posteriori* dietary pattern and the risk of | Healthy Spanish adults (M = 15,632, F = 25,806, age range 29–69 years, mean BMI 28.3 kg/m^2^)/ 1992–2004 | Sociodemographic and dietary data collected at baseline and 3 year follow-up. Dietary data collected through a computerised dietary history questionnaire. End points non | 662 foods in the questionnaire were classified into 33 groups. Principle component analysis used to identify two dietary patterns were identified - an evolved MedDiet pattern and a | Adjusted analysis: no significant association between adherence to a Westernised dietary pattern and CHD risk. 2^nd^, 3rd, 4th, and 5th quintiles of MedDiet pattern adherence |
|  |  | CHD in middle-aged persons. |  | fatal and fatal coronary events determined at 3-year follow-up and by hospital discharge records, mortality registries and myocardial infarction registries over a median follow-up of  11 years. | Westernised pattern. Each participant was scored on their diet, such that higher scores indicated higher adherence to the respective dietary pattern. Food intake in grams presented, as well as nutrient intake for quintiles of the two dietary patterns. | score were associated with a ↓ risk of CHD compared to 1st quintile (HRs were 0.77, 0.64, 0.56, and 0.73, and 95% CI were 0.61–0.98, 0.50–0.83, 0.43–0.73 and 0.57–0.94 respectively). P values were <0.05 for quintiles 2 and 5, <0.01 for quintile 3, and <0.001 for quintile 4. |
| Studies Reporting The Grams of Foods in the Meddiet | | | | | | |
| Kromhout *et al.*, (1989) [6] | Cross-sectional study | To describe the food consumption patterns of the 16 cohorts enrolled in the SCS at baseline. | Healthy male adults aged 50–59 years.  East Finland: *n* = 30, West Finland: *n* = 30,  Montegiorgio: *n* = 35,  Crevalcore: *n* = 29,  Rome: *n* = 49, Slavonia: *n* = 24, Dalmatia: *n* = 24,  Velika Krsna: *n* = 21, Zrenjanin: *n* = 40, Belgrade: *n* = 41, Zutphen: *n* = 45, US railroad: *n* = 30, Crete: *n* = 31, Corfu: *n* = 37,  Tanushimaru: *n* = 24,  Ushibuka: *n* = 8 /1959–1971 | To assess baseline dietary intake, the men were asked to complete WFRs. In all but two cohorts, 7-days were completed (4 days were completed in Ushibuka, and 1 day in the US railroad). Data was recorded as edible raw food for all but 3 cohorts; for those three the prepared products were converted back to quantities of raw products in a standardised way. All foods were put into 15 homogenous food groups (with similar water and nutritional content). A 16th heterogeneous group was created for miscellaneous products such as sauces, herbs and spices. | The MedDiet was represented by the three Italian cohorts and the two Greek cohorts. In Greece, bread, olive oil, potato, fruit, fish and legume intake was high. Meat, dairy, cheese and other cereal intake was low. In Italy, bread and to a lesser extent other cereals and olive oil intake was high. Meat, cheese, vegetable and fruit intake was moderate, and legume and potato intake was low. Italians had a moderate dairy and egg intake. Railroad workers in Rome had the highest vegetable intake of any of the 16 cohorts, while Greeks from Crete had the second highest edible fat intake (almost entirely as olive oil). | Pearson correlation co-efficient between food intakes in the 1960’s and the 1970’s were performed. *r* values were high for all food groups, highest for milk and cheese, fruit, meat, fish, sugar products and edible fats, indicating little change in these intakes over that time period. Potatoes had an *r* value of 0.68 which was not significant (*p* > 0.05), while all other correlations were significant (*p* < 0.05). |
| Trichopoulou *et al.*, (1995) [7] | Prospective  cohort study | To assess the influence of  a specific dietary pattern on overall survival | Elderly Greek adults (M = 91, F = 91) Aged ≥70 years at enrolment/1988–1990 | Using a validated 190-item semi quantitative FFQ, dietary intake of elderly residents in three Greek villages was established. Smoking status was also | An 8-unit *a priori* score was used to describe the diet. The eight components included MUFA:SFA, ethanol consumption, vegetables | There were 53 deaths. Only 19% of the sample had two or fewer points, and 57% had four or more points as per the *a priori* score. There was  a 17% ↓ |
|  |  |  |  | ascertained. After approximately  5 years follow-up number of cases of death from any cause was determined. Dietary intake was scored *a priori* using an 8-point scoring system to determine adherence to a principally healthy Mediterranean diet. Cox’s proportional hazards model for diet score as a predictor of survival over time was used to determine the relationship between adherence to the MedDiet and mortality. | legumes, cereals, fruits, meat and meat products and dairy products including milk. Sex-specific median values were used as the cut-off. One point was awarded for an intake above the cut-off for each of the following: MUFA:SFA, vegetables, legumes, cereals, fruits and ethanol. No points were awarded if intakes were below the median. One point was awarded for intakes below the median for meat and meat products and dairy. No points were awarded for intakes above the median for these two groups. | risk of mortality for each 1 unit ↑ in diet score (RR 0.83, CI 0.69–0.99, *p* = 0.04). For each 20 g ↑ in dairy, there was a 4% ↑ risk (RR 1.04, CI 1.01–1.07, *p* = 0.01, adjusted for energy intake). There were no other relationships between food groups, although MUFA:SFA ratio and legume intakes were associated with ↓ risk of mortality that did not reach significance (*p* = 0.14 and 0.13, respectively). Age was a strong predictor of death. |
| Kouris-Blazos  *et al.*, (1999) [8] | Prospective cohort study | To examine whether (our) results from the study in rural Greece (Trichopoulou  *et al.*, 2005) could be replicated in an urban setting in Australia, and to examine whether the apparent benefits of the MedDiet are transferable to population groups with very different dietary habits. | Elderly Australian adults, 141 Anglo-Celts (M = 70 and F = 71) and 189 Greek-Australians (M = 94, F = 95)/1990–1992 | A validated 250-item FFQ was used to ascertain dietary habits at baseline. Smoking status was also ascertained. After approximately 5 years follow- up number of cases of death from any cause was determined. Dietary intake was scored *a priori* using an 8-point scoring system to determine adherence to a principally healthy Mediterranean diet. Cox’s proportional hazards model for diet score as a predictor of survival over time was used to determine the relationship between adherence to the MedDiet and mortality. | MDS used, described above. (Trichopoulou *et al.*, 1995). | There were 24 Greek and 14 Anglo-Celtic deaths. Eighty-one per cent of Greeks had at least 4 points as per the *a priori* score, compared to 28% of Anglo-Celts. There was a 17% ↓ risk of mortality for each 1 unit increase in diet score (RR 0.83, CI 0.67–1.02, *p* = 0.07, adjusted for energy intake). Only fruit and nut and cereal intakes were independently associated with a ↓ risk of mortality, such that for each 20 g increase, there was a 5% (RR 0.95, CI 0.91–1.1) and an 8% (RR 0.92, CI 0.85–0.99) ↓ risk, respectively. |
| Trichopoulou  *et al.*, (2003) [9] | Prospective  cohort study | To investigate the relation of the Mediterranean dietary pattern and the MDS with overall mortality in a large sample of the general Greek population | Healthy Greek adults (*n* = 22,043)  Age range 20–86 years  Baseline BMI 28.1 kg/m^2^ for men and 28.8 kg/m^2^ for women/1994–1999 | Dietary intake was assessed using a semi-quantitative 150-item FFQ specific for Greece. Participants were followed for an average of 3.7 years. Date and causes of death was ascertained in this time. Primary outcomes were mortality from all causes, from CHD and from cancer. Data was summarised into 14 all-inclusive groups, and intakes in grams was calculated. Along with an updated MDS which included fish, intakes of the 14 food groups was associated with primary outcomes using Cox proportional-hazard regression models. | The 14 food groups included potatoes, vegetables, legumes, fruits and nuts, dairy products, cereals, meat, fish, eggs, MUFA, PUFA, SFA and margarines, sugar and sweets and non-alcoholic beverages. The updated MDS included the eight previous components and fish as a 9th group (Trichopoulou *et al.*, 1995). Additionally, points were awarded for an ethanol intake between 10–50 g for men and 5–25 g for women—outside these ranges, no points was awarded. | In general, the population studied had a high adherence to the MedDiet according to the MDS. The only individual dietary components significantly associated with total mortality was fruits and nuts (HR 0.82, CI 0.70–0.96) and the MUFA:SFA (HR 0.86, CI 0.92–0.98). A 2-point increment in the MedDiet score was associated with a 25% ↓ risk for total mortality (HR 0.75, CI 0.64–0.87, *p* < 0.0001, fully adjusted model). For CHD, the HR was 0.67 (CI 0.47–094) and for cancer the HR was 0.76  (CI 0.59–0.98). |
| Alberti-Fidanza *et al.*, (2004) [10] | Descriptive study | To assess how close or far the food intakes of population groups are from  a reference  dietary pattern. | Healthy Italian teenagers and adults (M = 158, F = 170). Dietary intake of adult population only reported (M = 121,  F = 125)/1960 | The diet of 40–59 year old men from rural Italy recorded in 1960 was used as a reference diet (Italian-Mediterranean). The dietary intakes of the men from the two Italian cohorts in the SCS (Crevalcore and Montegiorgio) were compared to this reference diet using the MAI at baseline (1965), and two follow-up points (1970 and 1991). The diets of the young relatives of the men were also compared using the index in 1991. The diets of groups from three other Italian regions were also analysed, including Pollica and Perugia. | The MAI is calculated by summing the percentage of total energy from Mediterranean foods (breads, cereal, legumes, potatoes, vegetables, fruit, fish, red wine, vegetables oils), and dividing this by the sum of the percentage energy from non-typical Mediterranean foods (milk, cheese, meat, eggs, animal fats, margarines, sweet beverages, cakes, pies, cookies and sugar). | For the reference diet, the 25th, 50th and 75th percentiles had a MAI of 5.4, 7.5 and 10.8 respectively. For Crevalcore, at baseline the MAI was 2.2, 2.9 and 4.4, and in Montegiorgio 4.0, 5.6 and 7.6 for the 25th, 50th and 75th percentiles respectively. In both cases the MAI scores were lower in 1970 and in 1991, showing a decline in adherence to the MedDiet in Italy over this time. In 1991, in Crevalcore the scores were 1.7, 2.2 and 3.0, and in Montegiorgio they were 2.6, 3.9 and 4.8 for the 25th, 50th and 75th percentiles, respectively. A |
|  |  |  |  |  |  | similar trend was observed for the populations of Pollica and Perugia between 1967 and 1999. |
| Vincent-Baudry *et al.*, (2005) [11] | Randomised controlled trial | To evaluate the effect of a Mediterranean-type diet compared with that of a low-fat (prudent) diet on CVD risk factors in subjects at moderate  CVD risk. | At-risk French adults (M = 82, F = 130)  Mean age 50.8 years in the Med group and 51.6 years in the low fat groups. Volunteers had to have one of the following: high fasting total cholesterol, triglycerides or BP, overweight (BMI > 27 kg/m^2^), be a smoker, sedentary or have a family history of CVD/1998–2002. | In a parallel design, volunteers were assigned to either a MedDiet or the low fat diet of the AHA. At baseline, sociodemographic data were collected, including physical activity and psychological state. Fasting measures were taken at baseline, three months and 12 months including the following: BMI, W:H, BP, intima-media thickness, total, HDL and LDL cholesterol, triglycerides, glucose, insulin, homocysteine, Apo-AI, Apo-B, Apo-B48, Apo-CIII, Apo-E, HOMA, inflammatory markers, plasma fatty acids, carotenoids, phenolic compounds, folic acid and vitamin B_12_. | The MedDiet intervention comprised of: 50% energy from CHO, mainly complex, 12%–15% energy from protein, 35%–38% energy from fat, a MUFA:SFA > 1, PUFA:SFA ≥ 0.5, SFA 8%–10% of total energy, PUFA 8%–10% of total energy, 18%–20% energy as MUFA, ≤300 mg cholesterol, >25 g fibre, 100 mg vitamin C, 7 mg carotenoids and ≤4% energy as alcohol in women, and ≤5% energy as alcohol in men. The low fat diet intervention was similar: 55%–60% energy from CHO, 15% energy from protein, 30% energy from fat, MUFA 10% of energy, >20 g fibre were the main differences. | Dietary changes were not significantly different between groups. BMI in both groups ↓ significantly  (−1.5 kg/m^2^ in Med group, −1.2 kg/m^2^ in the low fat group, *p* < 0.05). Plasma total cholesterol ↓ by 0.4 mmol/L in the Med group and 0.3 mmol/L in the low fat group (P for difference 0.082). LDL cholesterol followed a similar pattern to total cholesterol, ↓ by 0.5 mmol/L in the Med group and 0.2 mmol/L in the low fat group (P for difference 0.074). There was no change in HDL cholesterol, Apo-E or Apo-CIII after 3 months in either group. There was a significant decrease in both groups after 3 months for triglycerides, Apo-AI, Apo-B, glucose, insulin or HOMA score, but no between-group differences. |
| Studies Reporting the Grams of Foods in the Meddiet | | | | | | |
| Kromhout *et al.*, (1995) [12] | Retrospective cohort study | To study the associations between average intake of individual fatty acids | Greek, Italian, Finnish, Yugoslavian, Japanese, North American and Dutch adult male | Dietary data collected from small subsamples from 16 cohorts in the form of WFRs between 1959 and 1971. In | There were three Italian and two Greek cohorts representing the traditional Mediterranean diet. The records of the Italians were | The specific fatty acids were calculated for each cohort. Cretans had the highest intakes of oleic acid. Japan and Greece |
|  |  | and dietary cholesterol in the 16 cohorts in relation to average serum cholesterol and 25-year population mortality rates from CHD. | (age range 40–59 years)/1958–1964 | 1987, diets were recreated using foods collected from local markets (mean intake in grams of key foods replicated). The foods were homogenized and stored at −20 °C. The total lipid content including trans fatty acids, omega-3 PUFAs and saturated, MUFA and PUFA was determined by gas chromatography. Correlation coefficients were calculated for intakes of specific fatty acids and mortality from CHD, and cholesterol levels and mortality from CHD. | taken in 1960 and 1969 and in Greece between 1960 and 1965. The participants mostly lived in rural areas excepting an Italian cohort in Rome. | had the lowest rates of CHD after 25 years follow up. Saturated fat intake was significantly associated with mortality from CHD, particularly lauric (12:0) and mystic (14:0) acid (correlation coefficients 0.84 and 0.86, *p* values < 0.001, respectively). Intake of oleic and linoleic acid, and EPA and DHA was not associated with 25-year mortality. |
| Kafatos *et al.*, (2000) [13] | Descriptive article. | To describe the traditional diet of Crete and evaluate the nutrient composition of 3 types of diet common in Crete by means of chemical analysis of composite food samples. | NA | A 7-day menu for 3 typical Greek diets (traditional Cretan (Diet A), modern diet of Greek adolescents (Diet B), fasting diet of the Greek orthodox church (Diet C) was formulated. All foods in each menu were prepared normally, blended together and kept at −30 °C until analysis. The three menus were analysed for the nutrient content and compared with calculated nutrient content output from the Greek Diet database. | Diet A: menu reconstructed from the 7-day WFR’s kept by a 15-person subsample of Cretan men involved in the SCS in the early 1960’s. Diet B: menu based a dietary survey conducted amongst secondary school students in 1994 in Crete. Diet C: menu obtained from a 7-day WFR kept by a nun during the week before Palm Sunday in 1996. | Diet A was highest in total and monounsaturated fat, linoleic acid, fibre, alcohol, phytosterols, potassium, folic acid, vitamin A, vitamin E, vitamin C, vitamin B-6 and B-12. Diet B was highest in energy, protein, saturated fat, trans fats, omega-3 and -6 fatty acids, carbohydrate, cholesterol, calcium and phosphorus, and lowest in fibre, iron, oleic acid, potassium, magnesium, folic acid, vitamin E, and vitamin E. Diet C was lowest in energy, protein, fat and carbohydrates but nutrient rich. |
| Singh *et al.*, (2002) [14] | Randomised controlled intervention trial. | To assess the effect of an Indo-Mediterranean diet | High-risk Indian adults with either hypercholesterolemia, | Participants attended three weekly visits to obtain baseline clinical, dietary, laboratory and | Both groups were advised to follow the National Cholesterol Education Program step 1 | After 2 years, those in the intervention group consumed more calories from |
|  |  | consisting of whole grains including legumes, fruits, vegetables, nuts and mustard or soybean oil on fatal or non-fatal myocardial infarction, sudden cardiac death and the combined total of these events. | hypertension, diabetes mellitus or previous heart attack (age range 28–75 years, mean BMI 24.2 kg/m^2^)/dates data collection not reported | other data. Participants were randomised to receive either an Indo-Mediterranean diet or the control diet (National Cholesterol Education Program step 1 prudent diet). Follow-up was 2 years. Dietary and physical activity data was collected again at weeks 4, 8, 12 and 24 then at 12 week intervals. Laboratory data was collected again at 12 and 24 weeks then after 2 years follow-up. | prudent diet, with 30% energy from fat, <10% from saturated fat and <300 mg cholesterol per day. The intervention group received additional advice to consume 400–500 g fruits, vegetables and nuts, 400–500 g of whole grain cereals and legumes, and 3–4 servings of either mustard seed or soy bean oil per day. The intervention diet was intended to be high in phytochemicals, antioxidants, and α-linolenic acid. Weekly diet diaries were used with the aid of food models and household measures obtain nutrient intake. At all visits dietitians motivated participants to adhere to the dietary recommendations. | carbohydrate, had a higher PUFA:SFA, and consumed less energy, total fat, saturated fat and cholesterol. Those in the control consumed more n-3 fatty acids. Total and LDL cholesterol and triglycerides ↓ in both groups, significantly more so in the intervention group (*p* < 0.0001). In the adjusted model, there was a significant ↓ risk for non-fatal myocardial infarction (rate ratio 0.47, 95% CI 0.28–0.79), sudden cardiac death (rate ratio 0.33, 95% CI 0.13–0.86) and total cardiac endpoints (rate ratio 0.48, 95% CI 0.33–0.71) in the intervention group compared with the controls. |
| Ambring *et al.*, (2004) [15] | Randomised cross-over trial | To investigate whether a Mediterranean-inspired diet could beneficially affect vascular function. | Healthy Swedish adults (M = 12, F = 10, age 30–51 years, mean BMI 26 kg/m^2^/estimated between 1998–2002 | 4 weeks following a Mediterranean inspired-diet, 4 week wash-out period, 4-weeks following ordinary Swedish diet. Nutrient content reported based on 24-h recalls. | Rich in fruit, vegetables, fish, plant sterols and low GI carbohydrates. Similar energy, protein, carbohydrate and total fat to Swedish diet. | Total cholesterol ↓ 17%, LDL cholesterol ↓ 22%, triglycerides ↓ 17% and Apo-B↓ 16% after 4 weeks on MedDiet compared to Swedish diet (*p* < 0.05). No change in LDL particle size or endothelial function. |
| Trichopoulou  *et al.*, (2006) [16] | Review | To consider what a MedDiet is and how it affects longevity in Greece. To present evidence that the traditional Greek MedDiet is compatible with guidelines for a | NA | A weekly menu representing the traditional Greek MedDiet was analysed for the nutritional content (macro- and micronutrients) (further details not provided). | The menu was based on the Greek dietary guidelines, and is inclusive of both men and women, and respects religious practices (no animal products on Wednesday and Friday). Portion sizes were defined by Greek market regulations. It contained | The diet is comparable to European guidelines for a healthy diet. The diet is high in MUFA, fibre, relatively high in energy, and provides high amounts of micronutrients potassium and magnesium, plant sterols, vitamin E, and is |
|  |  | healthy diet. |  |  | regular fruit, fish, salad, olive oil, grains and nut consumption, little red meat and minimal processed food. | low in saturated fat. |
| Estruch *et al.*, (2013) [17] | Randomised controlled intervention trial | To test the efficacy of two MedDiets compared with a control diet on primary CVD prevention. | At risk, older Spanish adults; no CVD, and either Type 2 Diabetes, or three of the following: smoking, hypertension, elevated LDL, low HDL, overweight or obesity, family history of premature CHD.  Age range 55–80 for M, 60–80 for F, mean BMI 29.9 kg/m^2^/2003–2009 | Parallel groups followed either a low fat diet, a MedDiet supplemented with nuts or a MedDiet supplemented with EVOO for a mean follow-up of 4.8 years. Primary end point was combined myocardial infarction, stroke and death from CVD. | MedDiet groups asked to consume olive oil, nuts, fresh fruits, vegetables, fish, legumes, white meat and wine with meals often, and to limit soda drinks, bakery goods, margarines/butter and red and processed meats. EVOO group asked to consume 50 g EVOO per day. Nuts group asked to consume 30 g/day of mixed walnuts, almonds and hazelnuts. | Adjusted analysis: HR for primary end point 0.71 (95% CI 0.56–0.90, *p* = 0.004) (both MedDiet groups combined). Adjusted analysis for secondary end points: HR for stroke 0.61 (95% CI 0.44–0.86, *p* = 0.005), for myocardial infarction 0.77 (95% CI 0.52–1.15, *p* = 0.20), for death from CVD 0.83 (95% CI 0.54–1.29, *p* = 0.41) and for death from any cause 0.89 (95% CI 0.71–1.12, *p* = 0.32). |
| Studies Reporting the Flavonoid Content | | | | | | |
| Vasilopoulou  *et al.*, (2005) [18] | Descriptive article. | To calculate the intake of flavonoids from a traditional Greek plant-based weekly menu (using mainly the 2003 USDA flavonoid database). | NA | A weekly menu representing the traditional Greek Mediterranean diet was analysed for total flavonoid (flavones, flavonols, flavan-3-ols, flavanones, anthocyanidins and isoflavones) content using the 2003 USDA flavonoid tables, and the VENUS phytoestrogen database for isoflavones. Calculations for olives, dill and oregano were based on unpublished data, and calculations for herbal tea based on personal communication. | As Trichopoulou *et al.*, 2006. | The daily flavonoid intake was estimated at 118.6 mg, with flavanones contributing the most (32%), followed by catechins (28%), flavonols (22%), anthocyanidins (9%), flavones (8%) and isoflavones (1%). The largest source of flavanones was citrus fruits. Fruits, herbs, olives, onions and red wine were the greatest contributors to flavonoid intakes overall. |
| Dilis *et al.*, (2007) [19] | Descriptive article. | To analytically determine the flavone, flavonol, and flavan-3-ol content, and to compared the results with the respective theoretical flavonoid values of the same menu. | NA | A weekly menu representing the traditional Greek Mediterranean diet was analysed for the flavonoid content (luteolin, apigenin, myricetin, quercetin, kaempferol, isorhamnetin, catechin, epicatechin, epigallocatechin, epigallocatechin gallate, epicatechin gallate). All foods in the menu were prepared according to normal customs. Each day was combined to form a composite sample. Solids and liquids for each day were kept separate. | Same as Trichopoulou *et al.*, 2006 and Vasilopoulou *et al.*, 2007. | The average combined content of the flavonoids analysed was 79.01 mg/day. Nearly half (47%) was as flavonols (myricetin, quercetin, kaempferol and isorhamnetin). Another 40% of the total was as flavan-3-ols (catechins). Chemically determined content was higher than estimated content based on USDA flavonoid tables (67.8 mg compared to 79.01 mg). |
| Zamora-Ros  *et al.*, (2010) [20] | Cross-sectional study | To estimate dietary flavonoid intake and main food sources in a Mediterranean population (Spanish adults). | Healthy adults (M = 15,446 M, F = 25,237)  Age range 35–64 years  Spain/1992–1996 | Dietary data collected via a computerised dietary questionnaire was analysed for total flavonoid intake using the 2007 USDA flavonoid database. The dietary questionnaire contained over 600 foods and beverages and 150 recipes, and was administered by a dietitian. The USDA database for flavonoids was the most complete and up-to-date source for estimating dietary flavonoids at the time of publication. | The population studied was a sample of the Spanish population from five regions, two southern and three northern. The Spanish population typically followed a traditional Mediterranean diet. Data was collected between 1992 and 1996 and dietary intakes were comparable to the greater population at the time. | The mean daily flavonoid intake was calculated as 313.26 mg/day, and the median was 269.17 mg/day. Contributions were as follows: proanthocyanidins (60.1%), flavanones (16.9%), flavan-3-ols (10.3%), flavonols (5.9%), anthocyanidins (5.8%), flavones 1.1%) and isoflavones (<0.01%). Fruits, particularly apples and oranges, red wine, vegetables, chocolate and tea were the greatest contributors. |
| Tresserra-Rimbau *et al.*, (2014) [21] | Randomised controlled dietary intervention trial. | To evaluate the associated between the intake of total polyphenols and polyphenol subgroups and the risk of overall | At risk older adults; no CVD, and either Type 2 Diabetes, or three of the following: smoking, hypertension, elevated LDL, low HDL | Parallel groups followed either a low fat diet, a MedDiet supplemented with olive oil or a MedDiet supplemented with EVOO for a mean follow-up of 4.8 years. Primary end point | MedDiet groups asked to consume olive oil, nuts, fresh fruits, vegetables, fish, legumes, white meat and wine with meals often, and to limit soda drinks, bakery goods, margarines/butter | At baseline, higher polyphenol intake was associated with closer adherence to the MedDiet, more physical activity, and higher intake of alcohol and lower prevalence of |
|  |  | mortality. | overweight or obesity, family history of premature CHD. Age range 55–80 for M, 60–80 for F. Mean BMI 29.9 kg/m^2^ Spain/2003–2009 | was combined myocardial infarction, stroke and death from CVD. Polyphenol intake specifically rather than diet was used as the exposure. Dietary polyphenol intake was determined based on FFQs using the Phenol-Explorer database. Baseline characteristics were separated into quintiles of polyphenol intake. | and red and processed meats. EVOO group asked to consume 50 g EVOO per day. Nuts group asked to consume 30 g/day of mixed walnuts, almonds and hazelnuts. | hypertension. Hypercholesterolemia and smoking were more common among those with high polyphenol intakes. The hazard ratio was 0.60 (CI 0.39–0.91, *p* = 0.07) for total mortality for the highest quintile of polyphenol intake *versus* the lowest (fully adjusted for confounders, stratified by sex, centre and intervention group). |
| Studies Presenting a Meddiet Pyramid | | | | | | |
| Ministry of Health and Welfare (1999) [22] | Dietary guidelines for adults in Greece. | NA | Greek adults Greece/1998 | The FBDG of Greece were developed based on the traditional Greek dietary pattern, with incorporation of recent evidence from studies. The FBDG meet the Average Requirement for each nutrient. The guidelines are semi-quantitative, with a recommendation for number and size of servings. | The pyramid recommends daily cereals (non-refined), fruits, vegetables, olive oil and dairy products. Wine is also recommended in moderation along with daily physical activity. Fish, poultry, olives, pulses and nuts, potatoes, eggs and sweets are recommended weekly. Red meat is recommended 4 times per month. | NA |
| Oldways Preservation and Exchange Trust (2009) [23] | Diagram from webpage | NA | NA | NA | This pyramid recommends that every meal be based on one or more of the following: vegetables, seeds/nuts, legumes, fruits, grains (mostly whole), olive oil and herbs and spices. Fish and seafood should be consumed twice a week at least, poultry, dairy and eggs moderately, and meats and | NA |
|  |  |  |  |  | sweets less often. The pyramid suggests physical activity and social eating are important, to drink water and wine in moderation. |  |
| Bach-Faig *et al.*, (2011) [24] | Descriptive study | To present the MedDiet pyramid: a lifestyle for today | NA | The MDF in conjunction with the Forum on Mediterranean Food Cultures developed a consensus position to design a newly revised MedDiet pyramid. The pyramid is the culmination of scientific evidence and expert opinion, and promotes the consumption of fresh, minimally processed food but with a focus on portion size and moderation. It also aims to address availability, sustainability, accessibility and cost of foods and be adaptable to different cultures. | The pyramid represents a traditional MedDiet. It recommends physical activity, adequate rest, biodiversity, water consumption as well as recommendations for servings of food groups. The pyramid recommends fruits and vegetables, olive oil and bread and other wholegrain cereals every meal. Olives, seeds, nuts, herbs, spices and dairy should be consumed daily. White meat, fish, eggs, legumes, red meat, processed meat and potatoes should be consumed weekly, and sweets no more than twice weekly. | NA |

↑, increase; ↓, decrease; CVD, cardiovascular disease; BMI, body mass index; FFQ, food frequency questionnaire; MedDiet, Mediterranean diet; CI, confidence interval; CHD, coronary heart disease; EPIC, European Investigation into Cancer and Nutrition; M, Males; F, Females; FCS, food consumption survey; WFR, weighed food record; SCS, Seven Countries Study; HOMA, homeostasis model assessment; LDL, low-density lipoprotein; HDL, high density lipoprotein; CRP, C-reactive protein; HR, hazard ratio; MUFA, monounsaturated fatty acids; SFA, saturated fatty acids; RR, rate ratio; MDS, Mediterranean diet score; PUFA, polyunsaturated fatty acids; MAI, Mediterranean Adequacy Index; BP, blood pressure; AHA, American Heart Association; W:H, waist to hip ratio; Apo, apoplipoprotein; EPA, Eicosapenaetanoic acid; DHA, docosahexaenoic acid; NA, not applicable; EVOO, extra virgin olive oil; USDA, United States Department of Agriculture; FBDG, food based dietary guidelines; AR, average requirement; MDF, Mediterranean Diet Foundation.

**Table S2**. Full list of nutrient content presented by studies defining the MedDiet, intakes presented as total per day.

|  | **De Lorgeril *et al.* (1994) [25]** | **Kafatos  *et al.*, (2000) [13]** | **Ambring *et al.*, (2004) [26]** | **Trichopoulou *et al.*, (2006) [16]** | **Buckland  *et al.*, (2009) [2]** | **Itsiopoulos *et al.*, (2011) [4]** | **Guallar-Castillon  *et al.*, (2012) [5]** | **Estruch *et al.*, (2013) [8] Olive Oil ^a^** | **Estruch  *et al.*, (2013) [17] Walnut ^b^** | **Average Estruch  *et al.*, (2013) [17]** | **Average** | **SD (Where More than 1 Report)** |
| --- | --- | --- | --- | --- | --- | --- | --- | --- | --- | --- | --- | --- |
| Energy (kJ) | 8146.2 ^d^ | 11016.5 ^d^ | 7819.9 ^d^ | 10347.0 ^d^ | 8669.2 ^d^ | 9300.0 | 10020.7 ^d^ | 9087.7 ^d^ | 9326.1 ^d^ | 9206.9 ^d^ | 9294.0 | 973.87 |
| Total fat (g) | ND | 123 | ND | 110.7 | 82.5 ^e^ | ND | ND | ND | ND | ND | 105.4 | 20.78 |
| Saturated fat (g) | ND | 25 | ND | 29.8 | 22.6 ^e^ | ND | ND | ND | ND | ND | 25.8 | 3.67 |
| MUFA (g) | ND | 67 | ND | 63.8 | 37.5 ^e^ | ND | ND | ND | ND | ND | 56.1 | 16.19 |
| PUFA (g) | ND | 18 | ND | 9.9 | 11.8 ^e^ | ND | ND | ND | ND | ND | 13.2 | 4.23 |
| n-3 FAs (g) | ND | 0.7 | ND | ND | ND | ND | 2 | 0.9 | 0.8 | 0.85 | 1.1 | 0.54 |
| n-3 FAs (% energy) | 0.81 | ND | 2 | ND | ND | ND | ND | ND | ND | ND | 1.4 | 0.84 |
| Protein (g) | ND | 77 | ND | 74.5 | 90.8 | ND | 110.2 | ND | ND | ND | 88.1 | 16.37 |
| Carbohydrates (g) | ND | 294 | ND | 255.8 | 221.1 | ND | 254.2 | ND | ND | ND | 256.3 | 29.81 |
| Fibre (g) | 18.6 | 47 | 40 | 29.8 | 27.4 | 36.2 | 37.8 | 25.4 | 27 | 26.2 | 31.5 | 8.47 |
| Cholesterol (mg) | 203 | 123 | ND | ND | ND | ND | ND | 339 | 338 | 338.5 | 268.3 | 100.20 |
| Calcium (mg) | ND | 826 | ND | 696 | ND | ND | ND | ND | ND | ND | 761.0 | 91.92 |
| Magnesium (mg) | ND | 483 | ND | 234 | ND | 441 | ND | ND | ND | ND | 386.0 | 133.30 |
| Potassium (mg) | ND | 4504 | ND | 1774 | ND | 4565 | ND | ND | ND | ND | 3614.3 | 1594.07 |
| Sodium (mg) | ND | ND | ND | 2632 | ND | ND | ND | ND | ND | ND | 2632.0 |  |
| Iron (mg) | ND | 20 | ND | 14.9 | ND | ND | ND | ND | ND | ND | 17.5 | 3.61 |
| Phosphate (mg) | ND | 1322 | ND | ND | ND | ND | ND | ND | ND | ND | 1322.0 |  |
| Zinc (mg) | ND | 9 | ND | 10.3 | ND | ND | ND | ND | ND | ND | 9.7 | 0.92 |
| Folate (μg) | ND | 559 | ND | ND | ND | 453 | 512.5 | ND | ND | ND | 508.2 | 53.13 |
| Vitamin C (mg) | 115.8 | 258 | ND | ND | 172.7 | 191.1 | 279.9 | ND | ND | ND | 203.5 | 66.33 |
| Vitamin E (mg) | 11.6 | 17 | ND | 4.3 | ND | ND | 12.6 | ND | ND | ND | 11.4 | 5.27 |
| Sterols (mg) | ND | 267 | ND | 256.8 | ND | ND | ND | ND | ND | ND | 261.9 | 7.21 |
| Carotenoids (mg) | ND | ND | ND | 65.7 | ND | ND | ND | ND | ND | ND | 65.7 |  |
| Copper (μg) | ND | ND | ND | 3.8 | ND | ND | ND | ND | ND | ND | 3.8 |  |
| Manganese (mg) | ND | ND | ND | 3.51 | ND | ND | ND | ND | ND | ND | 3.5 |  |
| Alcohol (g) | ND | 17 | ND | 14 | ND | ND | 16.2 | ND | ND | ND | 15.7 | 1.55 |
| Vitamin A (mg) | ND | 20404 | ND | ND | ND | ND | 1306.5 | ND | ND | ND | 10855.3 | 13503.97 |
| Thiamin (mg) | ND | 2 | ND | ND | ND | ND | ND | ND | ND | ND | 2.0 |  |
| Riboflavin (mg) | ND | 1.8 | ND | ND | ND | ND | ND | ND | ND | ND | 1.8 |  |
| Niacin (mg) | ND | 18 | ND | ND | ND | ND | ND | ND | ND | ND | 18.0 |  |
| Pantothenic acid (mg) | ND | 5.1 | ND | ND | ND | ND | ND | ND | ND | ND | 5.1 |  |
| Vitamin B-6 (mg) | ND | 2.2 | ND | ND | ND | ND | ND | ND | ND | ND | 2.2 |  |
| Vitamin B-12 (mg) | ND | 2.5 | ND | ND | ND | ND | ND | ND | ND | ND | 2.5 |  |
| % Energy Fat | 30.4 | 42 | 34 | 39.6 ^f^ | 35.2 | 39 | ND | 41.2 | 41.5 | 41.4 | 38.2 | 4.10 |
| % Energy SFA | 8.0 | 8.4 | 8 | 10.7 ^f^ | 9.6 ^f^ | ND | ND | 9.4 | 9.3 | 9.4 | 9.1 | 0.91 |
| % Energy MUFA | 12.9 | 22.5 | 14 | 22.8 ^f^ | 16.0 ^f^ | ND | ND | 22.1 | 20.9 | 21.5 | 19.1 | 4.10 |
| % Energy PUFA | 4.6 | 6.0 | ND | 3.5 ^f^ | 5.0 ^f^ | ND | ND | 6.1 | 7.7 | 6.9 | 5.7 | 1.42 |
| % Energy Protein | 16.2 | 12 | 16 | 12.2 ^f^ | 17.8 | 13.5 | 18.7 ^f^ | 16.2 | 16.4 | 16.3 | 15.5 | 2.24 |
| % Energy CHO | ND | 45 | 48 | 39.6 ^f^ | 40.8 | 43.5 | 40.6 ^f^ | 40.4 | 39.7 | 40.1 | 42.0 | 2.92 |
| % Fat as SFA | ND | 9 | ND | 26.9 ^f^ | 27.4 | 8.2 | ND | ND | ND | ND | 17.9 | 10.72 |
| % Fat as MUFA | ND | 23 | ND | 57.6 ^f^ | 45.5 | 21.3 | ND | ND | ND | ND | 36.9 | 17.70 |
| % Fat as PUFA | ND | 6 | ND | 8.9 ^f^ | 14.3 | 6 | ND | ND | ND | ND | 8.8 | 3.92 |
| MUFA:SFA ratio | 1.61 ^f^ | 2.56 ^f^ | 1.75 ^f^ | 2.14 ^f^ | 1.66 ^f^ | 2.60 ^f^ | 2 | 2.35 ^f^ | 2.25 ^f^ | 2.30^3^ | 2.1 | 0.36 |

^a^ One intervention received a Mediterranean diet supplemented with extra virgin olive oil, dietary intakes for this group only presented; ^b^ One intervention received a Mediterranean diet supplemented with walnuts, almonds and hazelnuts, dietary intakes for this group only presented; ^c^ Average of the two intervention groups in the PREDIMED study presented. These figures were included in the final average total nutrients; ^d^ Converted from kcal by the following formula: (energy in kcal*4.184); ^e^ Calculated from per 1000 kcal, by the following formula: (nutrient content * total kCal/1000); ^f^ Calculated from gram values or percentages, not original provided. Abbreviations: SD = standard deviation. ND = no data. MUFA = monounsaturated fatty acids. PUFA = polyunsaturated fatty acids. n-3 FAs = omega-3 fatty acids.

**References**

1. De Lorgeril, M.; Renaud, S.; Mamelle, N.; Salen, P.; Martin, J.-L.; Monjaud, I.; Guidollet, J.; Touboul, P.; Delaye, J. Mediterranean Alpha-Linolenic Acid-Rich Diet in Secondary Prevention of Coronary Heart Disease. *Lancet* **1994**, *343*, 1454–1459.
2. Buckland, G.; González, C.A.; Agudo, A.; Vilardell, M.; Berenguer, A.; Amiano, P.; Ardanaz, E.; Arriola, L.; Barricarte, A.; Basterretxea, M.; *et al*. Adherence to the Mediterranean Diet and Risk of Coronary Heart Disease in the Spanish Epic Cohort Study. *Am. J. Epidemiol.* **2009**, *170*, 1518–1529.
3. Varela-Moreiras, G.; Ávila, J.; Cuadrado, C.; Del Pozo, S.; Ruiz, E.; Moreiras, O. Evaluation of Food Consumption and Dietary Patterns in Spain by the Food Consumption Survey: Updated Information. *Eur. J. Clin. Nutr.* **2010**, *64*, S37–S43.
4. Itsiopoulos, C.; Brazionis, L.; Kaimakamis, M.; Cameron, M.; Best, J.; O’dea, K.; Rowley, K. Can the Mediterranean Diet Lower Hba1c in Type 2 Diabetes? Results from a Randomized Cross-over Study. *Nutr. Metab. Cardiovasc. Dis.* **2011**, *21*, 740–747.
5. Guallar-Castillon, P.; Rodriguez-Artalejo, F.; Tormo, M.; Sanchez, M.; Rodriguez, L.; Quiros, J.R.; Navarro, C.; Molina, E.; Martinez, C.; Marin, P.; *et al*. Major Dietary Patterns and Risk of Coronary Heart Disease in Middle-Aged Persons from a Mediterranean Country: The Epic-Spain Cohort Study.
   *Nutr. Metab. Cardiovasc. Dis.* **2012**, *22*, 192–199.
6. Kromhout, D.; Keys, A.; Aravanis, C.; Buzina, R.; Fidanza, F.; Giampaoli, S.; Jansen, A.; Menotti, A.; Nedeljkovic, S.; Pekkarinen, M.; *et al*. Food Consumption Patterns in the 1960s in Seven Countries. *Am. J. Clin. Nutr.* **1989**, *49*, 889–894.
7. Trichopoulou, A.; Kouris-Blazos, A.; Wahlqvist, M.L.; Gnardellis, C.; Lagiou, P.; Polychronopoulos, E.; Vassilakou, T.; Lipworth, L.; Trichopoulos, D. Diet and Overall Survival in Elderly People. *BMJ* **1995**, *311*, 1457–1460.
8. Kouris-Blazos, A.; Gnardellis, C.; Wahlqvist, M.L.; Trichopoulos, D.; Lukito, W.; Trichopoulou, A. Are the Advantages of the Mediterranean Diet Transferable to other Populations? A Cohort Study in Melbourne, Australia. *Br. J. Nutr.* **1999**, *82*, 57–61.
9. Trichopoulou, A.; Costacou, T.; Bamia, C.; Trichopoulos, D. Adherence to a Mediterranean Diet and Survivial in a Greek Population. *N. Engl. J. Med.* **2003**, *348*, 2599–2608.
10. Alberti-Fidanza, A.; Fidanza, F. Mediterranean Adequacy Index of Italian Diets. *Public Health Nutr.* **2004**, *7*, 937–941.
11. Vincent-Baudry, S.; Defoort, C.; Gerber, M.; Bernard, M.-C.; Verger, P.; Helal, O.; Portugal, H.; Planells, R.; Grolier, P.; Amiot-Carlin, M.-J.; *et al*. the Medi-Rivage Study: Reduction of Cardiovascular Disease Risk Factors After a 3-Mo Intervention With a Mediterranean-Type Diet or a Low-Fat Diet. *Am. J. Clin. Nutr.* **2005**, *82*, 964–971.
12. Kromhout, D.; Menotti, A.; Bloemberg, B.; Aravanis, C.; Blackburn, H.; Buzina, R.; Dontas, A.S.; Fidanza, F.; Giampaoli, S.; Jansen, A.; *et al*. Dietary Saturated and Trans Fatty Acids and Cholesterol and 25-Year Mortality from Coronary Heart Disease: The Seven Countries Study. *Prev. Med.* **1995**, *24*, 308–315.
13. Kafatos, A.; Verhagen, H.; Moschandreas, J.; Apostolaki, I.; Van Westerop, J., J M. Mediterranean Diet of Crete: Foods and Nutrient Content. *J. Am. Diet. Assoc.* **2000**, *100*, 1487–1493.
14. Singh, R.B.; Dubnov, G.; Niaz, M.A.; Ghosh, S.; Singh, R.; Rastogi, S.S.; Manor, O.; Pella, D.; Berry, E.M. Effect of An Indo-Mediterranean Diet on Progression of Coronary Artery Disease in High Risk Patients (Indo-Mediterranean Diet Heart Study): A Randomised Single-Blind Trial. *Lancet* **2002**, *360*, 1455–1461.
15. Ambring, A.; Friberg, P.; Axelsen, M.; Laffrenzen, M.; Taskinen, M.-R.; Basus, S.; Johansson, M. Effects of a Mediterranean-Inspired Diet on Blood Lipids, Vascular Function and Oxidative Stress in Healthy Subjects. *Clin. Sci.* **2004**, *106*, 519–525.
16. Trichopoulou, A.; Vasilopoulou, E.; Georga, K.; Soukara, S.; Dilis, V. Traditional Foods: Why and How to Sustain Them. *Trends Food Sci. Technol.* **2006**, *17*, 498–504.
17. Estruch, R.; Ros, E.; Salas-Salvadó, J.; Covas, M.-I.; Corella, D.; Arós, F.; Gómez-Gracia, E.; Ruiz-Gutiérrez, V.; Fiol, M.; Lapetra, J.; *et al*. Primary Prevention of Cardiovascualr Disease With a Mediterranean Diet.
    *N. Engl. J. Med.* **2013**, *368*, 1279–1290.
18. Vasilopoulou, E.; Georga, K.; Joergensen, B., M; Naska, A.; Trichopoulou, A. the Antioxidant Properties of Greek Foods and the Flavonoid Content of the Mediterranean Menu. *Curr. Med. Chem.* **2005**, *5*, 33–45.
19. Dilis, V.; Vasilopoulou, E.; Trichopoulou, A. the Flavone, Flavonol and Flavan-3-Ol Content of the Greek Traditional Diet. *Food Chem.* **2007**, *105*, 812–821.
20. Zamora-Ros, R.; Andres-Lacueva, C.; Lameula-Raventós, R.M.; Berenguer, T.; Jakszyne, P.; Barricarte, A.; Ardanaz, E.; Amiano, P.; Dorronsoro, M.; Larranaga, N.; *et al*. Estimation of Dietary Sources and Flavonoid Intake in a Spanish Adult Population (Epic-Spain). *J. Am. Diet. Assoc.* **2010**, *110*, 390–398.
21. Tresserra-Rimbau, A.; Rimm, E.B.; Medina-Remón, A.; Martínez-González, M.Á.; López-Sabater, C.M.; Arós, F.; Fiol, M.; Ros, E.; Serra-Majem, L.; Pintó, X.; *et al*. Polyphenol Intake and Mortality Risk: a Re-Analysis of the Predimed Trial. *BMC Med.* **2014**, *12*.
22. Ministry of Health and Welfare, Supreme Scientific Health Council: Dietary Guidelines For Adults in Greece. *Arch. Hell. Med.* **1999**, *16*, 516–524.
23. Oldways Preservation and Exchange Trust. Mediterranean Diet Pyramid. Available online: http://Oldwayspt.Org/Resources/Heritage-Pyramids/Mediterranean-Pyramid/Overview (accessed on
    27 February 2013).
24. Bach-Faig, A.; Berry, E.M.; Lairon, D.; Reguant, J.; Trichopoulou, A.; Dernini, S.; Medina, F.X.; Battino, M.; Belahsen, R.; Miranda, G.; *et al*. Mediterranean Diet Pyramid Today. Science and Cultural Updates. *Public Health Nutr.* **2011**, *14*, 2274–2284.
25. De Lorgeril, M.; Renaud, S.; Mamelle, N.; Salen, P.; Martin, J.-L.; Monjaud, I.; Guidollet, J.; Touboul, P.; Delaye, J. Mediterranean Alpha-Linolenic Acid-Rich Diet in Secondary Prevention of Coronary Heart Disease. *Lancet* **1994**, *343*, 1454–1459.
26. Ambring, A.; Friberg, P.; Axelsen, M.; Laffrenzen, M.; Taskinen, M.-R.; Basus, S.; Johansson, M. Effects of a Mediterranean-Inspired Diet on Blood Lipids, Vascular Function and Oxidative Stress in Healthy Subjects. *Clin. Sci.* **2004**, *106*, 519–525.

© 2015 by the authors; licensee MDPI, Basel, Switzerland. This article is an open access article distributed under the terms and conditions of the Creative Commons by Attribution (CC-BY) license (http://creativecommons.org/licenses/by/4.0/).
